# Genomic Testing for Human Health and Disease Across the Life Cycle: Applications and Ethical, Legal, and Social Challenges

**DOI:** 10.3389/fpubh.2019.00040

**Published:** 2019-03-11

**Authors:** Gemma A. Bilkey, Belinda L. Burns, Emily P. Coles, Faye L. Bowman, John P. Beilby, Nicholas S. Pachter, Gareth Baynam, Hugh J. S. Dawkins, Kristen J. Nowak, Tarun S. Weeramanthri

**Affiliations:** ^1^Office of Population Health Genomics, Public and Aboriginal Health Division, Department of Health, Government of Western Australia, East Perth, WA, Australia; ^2^Office of the Chief Health Officer, Public and Aboriginal Health Division, Department of Health, Government of Western Australia, East Perth, WA, Australia; ^3^PathWest Laboratory Medicine, Sir Charles Gairdner Hospital, Nedlands, WA, Australia; ^4^Faculty of Health and Medical Sciences, School of Biomedical Sciences, The University of Western Australia, Crawley, WA, Australia; ^5^Genetic Services of Western Australia, King Edward Memorial Hospital, Department of Health, Government of Western Australia, Subiaco, WA, Australia; ^6^Faculty of Health and Medical Sciences, School of Medicine, The University of Western Australia, Crawley, WA, Australia; ^7^Western Australian Register of Developmental Anomalies, King Edward Memorial Hospital, Department of Health, Government of Western Australia, Subiaco, WA, Australia; ^8^Centre for Child Health Research, The University of Western Australia and Telethon Kids Institute, Perth, WA, Australia; ^9^Sir Walter Murdoch School of Policy and International Affairs, Murdoch University, Murdoch, WA, Australia; ^10^School of Public Health, Curtin University of Technology, Bentley, WA, Australia; ^11^Harry Perkins Institute of Medical Research, QEII Medical Centre, Nedlands, WA, Australia; ^12^Faculty of Health and Medical Sciences, School of Population and Global Health, The University of Western Australia, Crawley, WA, Australia

**Keywords:** genomics, public health, healthcare, genomic testing, molecular diagnostics, genetic disease, clinical utility, genomic data

## Abstract

The expanding use of genomic technologies encompasses all phases of life, from the embryo to the elderly, and even the posthumous phase. In this paper, we present the spectrum of genomic healthcare applications, and describe their scope and challenges at different stages of the life cycle. The integration of genomic technology into healthcare presents unique ethical issues that challenge traditional aspects of healthcare delivery. These challenges include the different definitions of utility as applied to genomic information; the particular characteristics of genetic data that influence how it might be protected, used and shared; and the difficulties applying existing models of informed consent, and how new consent models might be needed.

## Introduction

Genomic testing is used to diagnose, monitor, treat, predict and prevent disease, as well as promote good health in individuals, across communities and whole populations. Technological advances have allowed for greater integration of genomics into healthcare delivery, from screening and molecular diagnostics, to the accurate detection of microbes, and the ability to prescribe and monitor the efficacy of more precise therapeutics (1). The potential for increased use of genomic testing in the health setting is available throughout the life cycle, including in preimplantation, prenatal, neonatal, pediatric, adult, preconception, and posthumous settings (2). The person (who is often, but not always, also the “patient”) should be firmly at the center of the genomics revolution in healthcare. We begin this review by discussing a variety of current and emerging situations in which genomic testing is being utilized in health settings, focusing on the ethical, legal and social issues that apply at each point in the cycle-of-life and at particular decision points relevant to specific healthcare situations.

Subsequently, we focus on three main areas in which genomic technology, which is considered to be both disruptive and transformative to healthcare delivery (3), creates unique ethical issues that can challenge traditional aspects of healthcare. We summarize some of the key challenges and considerations surrounding the increasing application of this technology, highlighting issues that may arise when genomic tests are used at different life cycle stages. Within this section, we firstly outline the juxtaposition between clinical utility of a genomic test with personal and other utility, particularly where genomic testing is utilized in non-clinical settings (4). Related to questions around the utility of testing are issues surrounding the limited ability to interpret incidental findings and variants of unknown significance, which presents ethical challenges for the responsibility to return such results to patients (5).

Secondly, we consider how the personal nature of genomic data is such that it can never be truly de-identified. This creates potential issues around data storage and sharing; however, integral to this is the necessity to share genomic information to allow for advancement of knowledge of the etiology of disease (6). Furthermore, appropriate reference genomes are critically important for capturing the genomic diversity of the population being tested (7) so as to deliver equitable healthcare.

Finally, we discuss how genomic testing can challenge traditional models of informed consent in an environment where online DNA tests are available, where genomic testing is being increasingly utilized for individuals who are unable to consent, and where re-interrogation of stored genomic data is possible (8). For the purposes of this review, the term “genomics” is used to encompass both genetics (individual genes) and genomics (all genes in a genome).

## Current and Emerging Applications of Genomic Testing Across the Life Cycle

Genomic testing in the healthcare context can be applied in a multitude of ways throughout the human lifespan (Table 1). The application of massively parallel sequencing is expanding across different healthcare domains. This technique allows for the concurrent sequencing of numerous DNA fragments, enabling multiple loci to be investigated at one time and consequently, more efficient and cost-effective genomic analysis. Most of the current and emerging uses of genomic healthcare technology involve analyses for screening, diagnostic or prognostic purposes; testing to guide and evaluate treatment options; and identification and tracking of human disease-causing pathogens. Furthermore, genomic technology is increasingly available outside of healthcare settings through personal genomics tests that may be accessed directly by consumers, also known as direct-to-consumer or personal DNA tests (13, 22). Genomic sequencing technology has provided numerous benefits, particularly the significant improvement in the provision rate of molecular diagnoses (22). Continued developments with massively parallel sequencing include greater sensitivity of detecting previously difficult disease-causing deletions (23) and growing ability to detect copy number variants (24).

**Table 1:** Current and emerging applications of genomic tests across the life cycle.

**Type of test**	**Description**
Diagnostic	Used to investigate the cause of an observed phenotype. Testing follows onset of patient symptomatology, a clinical discovery or a positive screening test. Can be performed any time from *in utero* through to old age, and can be applied postmortem or posthumously. May detect germline or somatic variants.
Microorganism genomics	Involves testing the genomes of organisms that interact with and influence human health. Enables understanding and tracing of infections, outbreaks and identification of genomic changes behind antimicrobial resistance. Emerging applications include investigation of human microbiomes [e.g., lung, gut (9, 10)] and their influence on immunity, drug interactions, and disease expression (11).
Newborn bloodspot screening	Screening of newborns using blood collected by the Guthrie (heel prick) test. It typically detects the increased likelihood of the newborn having one of a number of rare and serious genetic conditions for which clinical interventions are available. Screening assays are typically biochemical, with a second line genomic test subsequently applied (possibly in conjunction with a clinical assay) to confirm the disease diagnosis for some conditions. The number and types of conditions included in newborn bloodspot screening programs varies around the world (12).
Personal/online DNA tests/direct-to-consumer	Genomic tests available direct to consumers through companies, with services ranging from having little or no clinical oversight through to comprehensive genetic counseling and clinician sign-off options (13). e.g., Ancestry.com, 23andMe, Genome.One, Counsyl, Helix, and Color. Options include gene panels for carrier, newborn, and inherited cancers testing. Availability of these services varies internationally.
Pharmacogenomics	Screening for genetic variants that alter drug-response with the aim of informing drug dosages and regimens to improve drug efficacy and patient compliance, whilst reducing side effects and avoiding life-threatening reactions.
Predictive/presymptomatic	Performed to establish an at-risk individual's predisposition to the development of a condition prior to symptoms onset. Traditionally this type of predictive testing involves both pre- and post-test genetic counseling. Huntington's disease provides a prototypical model.
Preimplantation genetic diagnosis (PGD)	Screening embryos created via *in vitro* fertilization (IVF) procedures to select those without a particular genomic variant/s for subsequent implantation. This follows identification of increased risk of the embryo having a genetic condition via molecular diagnosis or carrier screening of the parent/s.
Preimplantation genetic screening (PGS)	Screening embryos created via IVF procedures to select those without an identified chromosomal anomaly. This technique arose as an embryo selection tool in combination with IVF for women of advanced maternal age or with a history of failed implantation in IVF, to attempt to improve implantation rates for IVF cycles (14).
Prenatal/antenatal screening	*In utero* screening of a fetus can guide reproductive choice, preparedness and early interventions. An expanding approach for prenatal screening of genetic conditions is non-invasive prenatal screening (NIPS). From 10 weeks gestation, NIPS can be used to screen for the same chromosomal conditions as the combined first trimester screening test, as well as additional chromosomal disorders, by direct analysis of cell-free fetal DNA circulating in maternal blood. Although NIPS is non-invasive and is more accurate screening tool for these genetic conditions (15, 16), it is currently greater in cost than the combined first trimester screening test. Women who opt for NIPS over combined first trimester screening are still recommended to have an ultrasound (17), as these may detect pregnancy/fetal abnormalities not screened for by NIPS.
Prognostic	Utilizes gene variant/s or expression information to predict disease progression, severity and outcomes, as well as optimize and monitor therapeutic interventions. May also predict adverse responses to treatments.
Reproductive carrier screening	Traditionally used to determine the carrier status of couples suspected to be at a higher risk of having a child with a recessive or X-linked genetic condition. This has included individuals with an ethnic background known to have a greater prevalence of certain genetic conditions (e.g., Tay-Sachs disease in the Ashkenazi Jewish population) (18), or based on family history (e.g., a family member, including a previous child, with cystic fibrosis) (19).
	Simultaneous “expanded” carrier screening for more than one recessive or X-linked condition has been facilitated through the use of gene panels (20). In many countries it is possible for individuals or couples, including those with only average risk, to access these tests through a user pays scenario.
	When a couple is determined to be at greater risk of their future children having a genetic condition/s, their options include averting the birth of an affected child by refraining from having children, PGD, prenatal diagnosis and subsequent termination of an affected fetus, adoption or the use of donor gametes; preparation for the arrival of a child with a given condition; and the early commencement of treatments or preventions to alleviate disease in an affected fetus/child.
Posthumous	Molecular autopsies can occur on post-mortem tissue for sudden unexplained death (SUD), including *in utero* death. For example, inherited arrhythmia syndromes identified through molecular diagnosis may be identified as the cause of death for some autopsy-negative sudden unexpected death patients (21). For *in utero* deaths where other clinical signs were evident, or even those that might have been predicted, posthumous testing can confirm a suspected diagnosis. Increasingly this is also being applied for fetuses, stillbirths, and neonatal deaths with multiple congenital anomalies.

Genomic technology has increasing potential to contribute transformative new treatments in the form of genetic therapy. Recently this was demonstrated in a research setting with the treatment *in utero* of 3 fetuses affected with X-linked hypohydrotic ectodermal dysplasia. In this study, prenatal intervention occurred in the successful delivery of a recombinant version of the previously absent protein (25). Also on the horizon is the potential to use circulating tumor DNA in the blood or urine to assist in the clinical diagnosis of cancer, potentially alleviating the current reliance on invasive tissue sampling of solid tumors (26, 27).

The potential for genomic technology to continually improve the health and wellbeing of the population across the life cycle is anticipated. Further genomic and associated phenotypic data are integral for the advancement of knowledge and interpretation of genomic variants, and in turn the understanding of disease risk and disease etiology to inform better healthcare (6, 28, 29). The benefits of genomic technology in healthcare go beyond the immediate improvements in diagnosis and treatment, where a diagnosis is the portal to best care, to contributions to general understanding about disease and health and informing appropriately targeted public health initiatives.

The future ability to utilize “big data,” not only for the incorporation of genomic information but also other “-omic” information (e.g., metabolomics, proteomics), epigenetic, phenotypic, environmental and personal data, will depend on data collection and sharing. Big data will allow more precise healthcare, specifically healthcare that is tailored to individuals (i.e., precision medicine), and will facilitate precision public health interventions tailored to genetically identified population subgroups (30, 31). Additionally, large, shared datasets are likely to become even more important to further knowledge about disease mechanisms for common and complex polygenic diseases (29).

## Challenges and Considerations for Genomic Applications Across the Life Cycle

As described above, application of genomic technology and new genomic knowledge is being applied across the life cycle in both clinical and wider healthcare settings. Importantly, although the genomic technology itself may remain fairly consistent across different applications, the scope of the test can differ in terms of who is tested, for whom healthcare decisions are made about, the types of tests available, the potential conditions that can be identified, the clinical information available and the potential consequences of false positives or false negatives (see Table 2). Moreover, the issues surrounding utility, incidental findings and informed consent may differ depending on the life cycle stage and purpose of the test (Table 2).

**Table 2:** The scope and challenges associated with genomic testing across different life cycle stages.

**Considerations across the life cycle**	**Life cycle stage**					
	**Reproductive age**	**Preimplantation**	**Prenatal**	**Pediatric**	**Adult**	**Posthumous**
**SCOPE OF TEST**						
Who is primarily tested?	Prospective parents	Embryo	Fetus/mother	Child	Adult	Deceased
Who does the healthcare decision primarily concern?	Prospective parents/potential embryos	Embryo	Fetus/mother	Child	Adult	Family members
Is a phenotype available at time of testing?	No	No	Possibly	Possibly	Possibly	Possibly
**TYPE OF TESTS AVAILABLE**						
Screening	✓	✓	✓	✓	✓	
Diagnostic		✓	✓	✓	✓	✓
Personal/direct-to-consumer (non-clinical)/pharmacogenomics				✓	✓	
Microorganism			✓	✓	✓	
Prognostic/Predictive/Presymptomatic		✓	✓	✓	✓	
**CONDITIONS IDENTIFIED**						
Inherited germline	✓	✓	✓	✓	✓	✓
Acquired germline		✓	✓	✓	✓	✓
Somatic				✓	✓	✓
**PERSONAL AND CLINICAL UTILITY OF GENOMIC INFORMATION FOR TESTED INDIVIDUALS AND/OR FAMILY MEMBERS VIA CASCADE SCREENING**						
Reproductive choice (e.g., not having children, assisted reproduction, termination)	✓	✓	✓	✓	✓	✓
Preparation for future	✓	✓	✓	✓	✓	✓
Prevention or intervention			✓	✓	✓	
Providing a molecular diagnosis (new or suspected)		✓	✓	✓	✓	✓
Inform treatment and/or management options			✓	✓	✓	
**POTENTIAL HEALTH CONSEQUENCES OF FALSE POSITIVES**						
Decision not to implant an unaffected embryo		✓				
Termination of an unaffected fetus			✓			
Unnecessary use of assisted reproductive technology	✓					
Over diagnosis, over treatment, or wrong treatment			✓	✓	✓	
Unnecessary cascade testing or cascade testing for a wrongly attributed variant	✓		✓	✓	✓	✓
**POTENTIAL HEALTH CONSEQUENCES OF FALSE NEGATIVES**						
Missed opportunity for prior preparation, prevention, or intervention	✓	✓	✓	✓	✓	✓
No or wrong treatment			✓	✓	✓	✓
Missed opportunity for cascade testing for a wrongly attributed variant	✓		✓	✓	✓	✓
**INFORMED CONSENT**						
Tested individual unable to consent		✓	✓	✓		✓
**ASPECTS RELEVANT ACROSS THE LIFE CYCLE**						
Implications, considerations, and uses of test results	Research translation, incidental or secondary findings, non-actionable findings, non-health related traits, forensic investigation, ancestry, insurance, variants of unknown significance, sensitivity of data, longevity of data, versatility of data, reference data, genomic literacy.					

For example, the ethical issues surrounding diagnostic testing are undoubtedly less contentious than those involved with predictive testing of a living individual. In the first scenario a patient has usually already presented to a healthcare professional with symptomology, therefore the choice of the genomic diagnostic test and subsequent data interpretation is simplified and clinical utility usually aligns well with an individual's expectation of personal utility. This is true even if a treatment or intervention is not available, as a molecular diagnosis can end the diagnostic odyssey and potentially facilitate access to applicable health and social services (32). Comparatively, it can prove more complicated to predict the likelihood of a person potentially developing a disease at some point in the future. This is especially true for a person who has not yet been born, such as when predicting risk of a disease for a current (preimplantation or prenatal diagnosis) or future (preconception carrier screening) embryo or fetus. The absence of familial information such as in the case of adoption or gamete donation can add more complexity. The potential implications of the action/s taken based on genomic information in these situations are also greater, due to the possibility of an embryo not being selected for implantation, or the choice to terminate an affected fetus (Table 2).

Despite the potential benefits associated with increasing use of genomic testing, our knowledge of the relationship between genomic variants and health is still evolving and is limited by the complex interactions between the genome and other biological and environmental influences. Three key considerations relating to this potential include the utility of genomic testing for clinicians and the person, or patient; issues around genomic data such as the sensitivity and potential longevity of the data, data sharing and appropriateness of the reference genome; and issues around informed consent in the context of the complexity and expanding usage of genomic testing. The issues that are explored below, while not exhaustive, provide a broad overview of some of the challenges and considerations of genomic testing across the life cycle. Additional ethical, legal and social issues associated with genomic testing, including challenges associated with direct-to-consumer testing, are beyond the scope of this paper and are discussed elsewhere (33, 34).

### Utility

#### Personal and Clinical Uses for Genomic Information

Clinical utility for genomic testing is generally limited to situations where genomic information directs and improves patient care. Therefore, genomic testing may not be recommended clinically if the results are unlikely to impact care. However, genomic information has multiple applications beyond the healthcare setting. For example, individuals may wish to have their genome sequenced to learn about their ancestry or non-health related traits; a genomic diagnosis can inform eligibility for special education and employment services; and genomic information is of interest to insurance companies and can be used in forensic settings. The manifold nature of genomic information could allow for more efficient healthcare due to the potential ability to reinterrogate data for multiple healthcare purposes. However, the value of such data to individuals and organizations beyond healthcare raises issues around consumer expectations when clinical utility and other kinds of utility diverge.

The utility of testing varies across the lifespan, potentially in relation to when the testing is performed and for which conditions the testing may relate (Table 2). In some settings genomic information is used to make healthcare decisions about an individual who may not have been able to provide consent (e.g., prenatal screening or diagnosis; patients with cognitive impairment). Furthermore, in particular settings the test not only informs treatment and management, but influences reproductive decisions which can be inherently more personal (as opposed to clinically led), compared to other healthcare decisions.

There are also limitations to the clinical utility of testing. For example in the preconception setting, the genomic test can only inform parents about risk of inherited conditions, and cannot provide information about *de novo* or acquired genetic conditions (Table 2). A particular consideration for the utility of testing across the life cycle is the presence (or absence) of a phenotype to aid clinical, as well as personal, decision-making. Genomic data has the potential to identify disease risk earlier than is possible through the use of clinical or physiological symptoms alone, meaning that interventions can be enacted more promptly. However, there is a conundrum presented whereby presence of a genetic variant may not necessarily mean that the disease will manifest (i.e., there is incomplete penetrance) (35), or where a variant is variably expressed among individuals and it is not possible to predict the severity based on genomic information alone.

In such situations, clinicians would ideally draw upon the presence of phenotype to aid in decision-making, and this is absent in the preconception and preimplantation stages, and often absent, or markedly limited, in the *in utero* setting (Table 2). Likewise, the use of genomic testing for screening includes the testing of asymptomatic individuals by definition, and this can occur at any life stage (Table 2). Assurance of the natural progression of genetic variants and the likelihood of resulting disease is required for individuals to make reproductive and healthcare choices. Similarly this information is needed for clinicians to make clinical decisions, yet currently this knowledge is limited. As a consequence, the clinical utility of genomic testing for many conditions currently remains relatively low.

In some circumstances, genomic information may have greater personal utility to patients compared to other medical test results. Therefore, there may be times where personal utility and clinical or healthcare utility are non-congruent. Available evidence indicates that many consumers would only seek genomic testing for actionable health information, particularly in situations where there is a perceived need for that information (4). However, certain subsets of the population may be more receptive to receiving non-actionable genomic results, in which case personal utility may override a lack of overt clinical utility. Common situations where these benefits arise include for parents of children with rare undiagnosed genetic diseases (36), testing for the risk of late-onset conditions at a point in the lifecycle where there is no clinical utility for such information (37), and testing for risk of conditions with no current treatments, such as Alzheimer's disease predisposition (38). Additional benefits cited for wanting to access testing in these situations include ending the diagnostic odyssey, a clearer understanding of the cause of a condition, greater understanding of future needs, the ability to connect with others in the same situation, and helping to gain access to social and healthcare services (32, 38, 39). Personal utility of a genomic test may also vary depending on a person's cultural background (40, 41).

In the era of personalized medicine and given that the degree of personal utility is likely to vary between persons, the utility of a genomic test is ultimately best estimated on an individual basis (42). Empowering patients and families to be involved in decisions will help to facilitate this (32). However, individuals and healthcare providers should recognize the inherent differences in value placed on genomic testing by different stakeholders. Variation in perceptions of the utility of genomic testing highlights the importance of accessible and appropriate education and genetic counseling for anyone considering a genomic test (43) (see Box 1). To enable this, sufficient genomic literacy is required across the health workforce to ensure health professionals are able to competently counsel individuals and families (46, 47). This could be enabled through increased education and training of health professionals (48).

Box 1 **Genetic counseling.**

Genetic counseling has traditionally focused on the education and support for patients and family members who have, or are at risk of, Mendelian disorders (44). With the increased integration of genomic testing in the healthcare system across the life cycle, the ability to provide adequate counseling to patients with the current workforce and services model will be tested. The current workforce will be challenged to provide optimal counseling with increased demand, and new models for delivering genetic counseling services may be required to fill this gap. New models could include bolstering the skills of the primary healthcare workforce (45), increasing the use of evidence-based online delivery, and utilization of technology such as telehealth to mitigate hurdles such as geography for the rural and remote population. Issues around genetic counseling for genomic tests are outlined in Burns et al. 2019 (46).

#### Research and Translational Uses of Genomic Testing

Genomic testing may be used in situations where the primary utility is to inform basic research or to identify eligibility for treatments or services. For genetic conditions without well-defined natural histories, for which there are few or no treatments available (e.g., rare diseases, including rare cancers), or where disease progression is rapid (e.g., advanced cancers), research and translational uses of genomic testing may be crucial. There could be a lack of knowledge or clinical consensus of the clinical utility of genomic testing in such contexts (49), but there might still be real or perceived utility for patients, clinicians, and researchers. Due to the rarity of genetic conditions and the increasing utility for genomic testing in treating cancer, management of these particular patient cohorts relies heavily on basic research.

Consideration of these additional uses for genomic testing in healthcare is important, particularly through the lens of providing equitable healthcare to those who need it. Only 14% of new scientific discoveries enter daily clinical practice, after an average lag of 17 years (50). This lag is especially problematic for (i) cancer patients, where disease prognosis can often be poor, but for whom tailored treatment is showing promise, and (ii) for patients with rare diseases for whom no, or limited pre-existing treatment is available. In this context, translating the benefits of genomic technology into the healthcare setting in a timely manner is challenging (51). Multiple agencies at both the national and international levels may be working on similar, smaller-scale research projects. This could lead to inconsistent findings due to sample size limitations, thereby becoming one of the rate-limiting steps in the integration of genomic advances into daily practice, and then into population-level programs. National and international collaboration between researchers has the potential to streamline this approach so that collective goals are expedited, and translation into practice is accelerated (51–53).

An additional consideration around the lag in translation of testing into the public healthcare setting is the potential for individuals to have their genome sequenced in a non-clinical setting (e.g., personal genomics) or a private healthcare setting, which may create expectations for increased access in public health systems. Genomic tests may be introduced first into private (user pays) settings due to the initially high cost and the lag in translation into public health systems, which must balance investment into multiple different aspects of healthcare (54), and as a result, often have a high evidence threshold for integrating new tests into practice. This can increase demand for, and produce inequity of access to, testing that may be beneficial for improving healthcare, but which health systems are not yet prepared to offer at a population-wide level (55).

#### Incidental or Secondary Findings

Genomic data can be interrogated in different ways, including broad approaches designed to capture as much information as possible and targeted approaches focused on a few variants of interest. Incidental or secondary findings are classified as gene variants that are not the primary focus of a specific genomic test and are not necessarily related to the condition/s being investigated [e.g., (56)]. These variants can be obtained when performing whole genome and exome sequencing, as well as with certain gene panels, and if scrutinized, may reveal the need to consider medical action. There have been efforts to identify a minimum list of variants that are considered medically actionable. The American College of Medical Genetics and Genomics has developed a widely used list of 59 genes to be reported in clinical settings as incidental or secondary findings (57). Incidental findings relating to germline variants and familial relationships are relevant across the life cycle (Table 2). A particular point of difference is the use of non-invasive prenatal testing, in which fetal DNA and maternal DNA can be identified in the same sample, which may reveal incidental findings relating to somatic changes present in the mother.

There is variation among consumers, pathologists, specialists, researchers and professional societies regarding the need to return incidental findings to patients, with the definition of incidental often being dependent on the medical specialty or setting (58, 59). Consumers may consider that raw data or medically significant incidental findings should be accessible to them even when their genome is sequenced for a non-clinical purpose (60, 61). This raises important legal issues surrounding the right to access personal information (43, 60), particularly when incidental findings relate to potentially actionable variants (62). However, there may be issues in the clinical utility of such information given the different requirements for sequencing quality in non-clinical settings (60, 63). Managing consumer/patient expectations is therefore of critical importance.

A dialogue involving all stakeholders, including the patient or consumer, may be required to agree on a set of criteria that could indicate when incidental findings should or should not be returned to individuals (64). A relevant question is whether researchers and genomics laboratories should be returning raw data or incidental findings given that many patients access genomic testing through research projects or directly from laboratories. Recent changes to the National Health and Medical Research Council's *National Statement for Ethical Conduct in Human Research*, with which researchers must comply, require a system to be in place to return findings that have health significance to participants (65). Other relevant questions include, will guidelines and regulations around incidental findings influence clinicians in ordering gene-specific analyses rather than clinical genomes or exomes? What is the likely effect of this on the utility of the test?

#### Variants of Uncertain Significance

By definition, variants of uncertain significance (VUS) have unknown clinical utility (66). It is understood that as knowledge of genomic variants and the relationship between variants and disease pathology advances, variants that have previously been identified as uncertain may be recognized as pathogenic, or alternatively benign. Internationally, guidelines surrounding the responsibility to report VUS are a work in progress. Variant interpretation, even in the setting of a clear phenotype, can be challenging at both the individual and health system levels. The medico-legal implications of variants at one point in time being identified as uncertain, and then with advances in knowledge being later identified as pathogenic, are highlighted in the law case of Williams vs. Athena (see Box 2). Significant questions include where do we draw the line on requirements to report based on knowledge of pathogenicity? How frequently should reference databases be updated and consulted to inform contemporary interpretation of results? Do patients wish to know about VUS if they are found? Is there an obligation to follow up VUS after the test has been taken, in accordance with changes in classification as knowledge develops? If so, who is responsible for doing this?

Box 2 **Case study-Legal issues in variant interpretation.**

A case study which highlights potential legal issues surrounding variant interpretation is the current USA lawsuit, *Williams* vs. *Athena*, where a 2 year old boy, Christian Jacob Millare, died of a fatal seizure from Dravet syndrome in January 2008. In 2007, Christian was tested for Dravet syndrome, and the report concluded that he had a “variant of unknown significance,” and as such Christian continued to be treated as if he had a mitochondrial disorder rather than Dravet syndrome. In 2015, the laboratory responsible for the test, Athena, updated its report to reclassify the mutation as pathogenic for Dravet syndrome, as Christian was found to have a pathogenic variant in the *SCN1A* gene. Interestingly at the time of the initial report, there were two publications (published in 2006 and 2007) each based on another single case reporting the same variant that Christian had as being pathogenic. Inherent to this case were the issues of whether a laboratory classifies as a health service provider, and relatedly the communication and responsibilities of laboratories and physicians. The case also emphasized the need for stricter governance around how variant databases report and interpret their data (6).

### Genomic Data

Genomic data are becoming increasingly useful not only for understanding the causes of ill health, but also the genomic determinants of good health. However, there are ethical, legal, and social issues that need to be considered to ensure appropriate use of genomic data in healthcare. These include that genomic information is not only personal, but also familial (i.e., it can reveal familial relationships and personal information about relatives); that the longevity, particularly of germline genomic data, exceeds that of typical health information; that the broad utility of genomic data increases the demand for data sharing; and that equitable genomic testing relies on appropriate reference data.

#### Sensitivity of Genomic Data

The collection and generation of genomic information comes with some unique ethical issues. The security and privacy of genomic information challenges traditional paradigms of confidentiality for sensitive information, given that a person's genomic information can be compared to a fingerprint, such that it can never be truly de-identified. Genomic information may therefore require additional regulation, such as the addition of noise to the data to ensure protection of privacy (67, 68).

Genomic data can impact biologically related family members even if they have not accessed genomic testing themselves. With the possibility for on-sharing of genomic information for future uses other than the intended purpose of the initial test, concerns over the privacy of genomic data and issues of informed consent for the disclosure and use of genomic information become paramount. Recently this was illustrated in California, where police used genomic information from an open source database from genealogy company GED match to facilitate the arrest of a serial rapist and murderer known as “The Golden State Killer” (69). The suspect, who is accused of committing crimes more than 3 decades ago, did not himself have genomic information in the database, but rather one of his relatives had participated.

#### Longevity of Genomic Data

Germline genomic data is somewhat unique in its unchanging nature, meaning that the same data could be reinterrogated over the life cycle for different health and non-health related purposes. The potential of genomic information is therefore longer lasting in contrast to many other types of health test results, which may provide more of a health snapshot. This makes genomic information more similar to stored biological samples collected in biobanks. Future innovations will allow for improved interrogation of historical genomic data (3, 70). In the inevitable advent of improved interrogation ability, is it ethical to analyse existing, historical datasets with improved technologies, and contrastingly, is it ethical to omit reinterrogation of existing datasets with new technologies (71)? Future tools for analysis and enhanced genomic knowledge may alter an individual's risk profile with reinterrogation of existing data (72). With increasing applications in precision medicine and precision public health, disease risk could become dynamically updated depending on current knowledge and data. The potential for rapid change in technology should be considered when determining how long to store genomic data. Careful management of consumer or patient expectations should also be considered.

The integration of genomic testing that is currently occurring separately across the life cycle may facilitate greater efficiencies in healthcare, but would require coordination and communication among previously isolated healthcare settings. With the increasing application of genomic testing across the life cycle, the potential to use one test for multiple purposes is increasing (73). For example, if genomic testing is introduced at a population level in more than one setting, the possible number of individual tests taken over the lifespan for a single individual is likely to increase. Assuming there is agreed utility for each type of testing at a population level, would broad sequencing such as whole genome or whole exome sequencing and long-term storage of genomic test results ultimately become more cost effective than repeat testing?

What if every baby's exome or genome was sequenced and reanalyzed as needed for different purposes throughout their life, would the cost savings from a reduced need to re-test outweigh the cost of storing the relevant data? A single genomic test could provide the answer to a patient's symptomatology that would have otherwise required multiple tests (74), and may also provide information relevant for different healthcare decisions in that person's life (Table 2). However, it may also lead to a short-term increase in healthcare demand from different healthcare professionals. For example, an actionable secondary finding may mobilize cascade screening in family members that would otherwise not have occurred (53), or may indicate consideration of alternative reproductive choices for individuals in the family. In addition, such long term storage and reinterrogation of genomic data would require processes for obtaining consent for each subsequent analysis over a long time period. The implications of broad sequencing methods and use of genomic data should be carefully examined prior to any large-scale implementation (75).

The potential for integration of genomic testing across the life cycle will not be without challenges. In such a context it is important to determine who is responsible for reporting results; which results should be reported and when; and whether to store and reanalyse results at a later time. The complexities of healthcare systems and governance arrangements across responsible organizations create the need for a harmonized approach at the local, national and international levels.

#### Data Sharing and Regulation

The benefits to be obtained from the use of genomic technology in healthcare are reliant on global cooperation and data sharing (2, 76). This is particularly the case for rare genetic conditions, where sharing of patient data can be critical to achieving a diagnosis or conducting research to improve healthcare. Adequate governance and regulation is required to ensure that genomic data are used responsibly by those who are permitted access. In 1997, the United Nations Educational, Scientific and Cultural Organization developed a Universal Declaration on the Human Genome and Human Rights, and, in 2003, adopted an International Declaration on Human Genetic Data to guide the use of such data at an international level (77).

Internationally, the regulatory space continues to evolve. The United States of America (USA) has developed regulations and legislation governing the protection and use of genetic information. The *Genetic Information Nondiscrimination Act of 2008* protects the genetic privacy of individuals by preventing insurers or employers from requesting genetic information. However, a bill for the *Preserving Employee Wellness Programs Act* has been introduced to the USA Senate, and has the potential to jeopardize present legislation protecting individuals from their employers requesting disclosure of genetic information and imposing penalties for non-disclosure (35). In Canada, the *Genetic Non-Discrimination Act* prohibits a person from requiring an individual to disclose their genetic information as a condition of providing goods or services to that individual, with exceptions for healthcare practitioners and researchers.

The requirements for responsible use of genomic information are similar to those for other health-related and personal information. The European Union (EU) has developed a General Data Protection Regulation that applies to businesses that process or control data from within the EU. Discrimination against individuals on the basis of their genetic information is also banned in Europe in accordance with the Council of Europe's Oviedo Convention. These regulations are important to ensure that data can be shared responsibly and ethically, which will encourage individuals to continue to participate in health research.

#### Reference Data

The importance of reference genomes in utilizing genomic testing is reliant on large-scale data sharing and collaboration. Reference genomes are used for comparison purposes, as a representative example of a typical human's genome sequence. These genomes are developed using a mosaic of genetic information sourced from different individuals and combined to form a template sequence. The use of reference genomes in utilizing genomic technology raises concerns around offering genomic testing that is less effective in some ethnicities compared to others, since genetic disease risk varies among ethnicities (78, 79). However, the use of an appropriate reference genome is made more difficult by the increasing multiculturalism within countries.

For equitable understanding of genomic variants, reference databases must be capable of reflecting the ethnic diversity of the relevant population/s, such as minority groups and/or Indigenous populations (80). It has been highlighted that most genomic reference databases do not adequately reflect the diversity of human populations (7). As an example, the cultural makeup of Australia's population is highly diverse, including Aboriginal and Torres Strait Islander peoples, and having a higher proportion of people born overseas compared to the USA, New Zealand, and Canada (81). However, there is a paucity of genetic data available for Aboriginal and Torres Strait Islander peoples, such that interpretation of genetic variants currently needs to be addressed with caution (82). For multicultural societies, it is vital to ensure that minority ethnic populations are not disadvantaged in accessing the benefits of genomic technology.

### Informed Consent

Informed consent for genomic testing in healthcare is complex when we try to consider all of the issues outlined above, including the variety of different testing methods and potential to test for multiple conditions at once; the testing of non-consenting individuals; potential for reinterrogation or sharing of data; the lack of diagnostic certainty in particular test settings; the complexities surrounding interpretation of tests, VUS and incidental findings; implications for insurance and other services; and the potential impact of testing on family members. Some of these issues are more pertinent in particular test settings across the life cycle (Table 2).

For example, the current guidelines on prenatal testing from the Royal Australian and New Zealand College of Obstetricians and Gynaeocologists state that information provided to women should include descriptions of conditions to be tested, as well as information about both the variance in phenotype and ability to predict the conditions tested (83). In the context of increasing lists of conditions to be screened for through the use of expanded genetic testing panels or whole exome/genome sequencing, how can healthcare providers ensure that the decision to undergo such testing is adequately informed?

The complexity of ethical, legal, and social issues surrounding consent for genomic testing indicate that substantial effort is required to ensure adequate understanding of the test by consumers. Depending on how many of these issues apply, professional genetic counseling may be critical for obtaining truly informed consent for genomic tests. Informed consent should be obtained based on the individual's circumstances to the extent that this is possible. Patient decision aids may be helpful for achieving this approach, particularly for applications where for some people the personal benefits outweigh the lack of clinical utility (84, 85). Such tools have been developed to help parents decide whether to undergo screening for Down syndrome (86), and to support reproductive decision-making for individuals with a genetic predisposition to heritable cancer (87). Decision aids may need to be tailored to the level of health literacy of users (88).

Consideration must also be given to a model where informed consent to personal genomic data analysis and storage can allow for the possibility that data may be reviewed, and thus for the possibility that an individual or their family might be contacted in relation to the updated outcomes in the future if this occurs (89). In the context of including late-onset conditions in tests performed on fetuses or children, is there a moral obligation to ensure this information can be communicated to individuals once they reach the age of informed consent? Dynamic consent models in this instance are one such example of a flexible, digitally-enabled consent model that may cater more broadly to the needs of healthcare consumers (8). Evidence so far shows that healthcare consumers want ownership and control over their health data (90, 91). Several companies are developing applications to allow greater access to, and control of, genomic information by consumers (e.g., Helix online genomics marketplace; Seqster platform). Much of this development is currently occurring in the personal genomics space, but similar efforts are being made in public health systems in association with moves to electronic health records. This patient-centered approach will be increasingly important with the rise in personalized medicine and precision public health, but will need to be implemented in a considered and ethical manner.

## Conclusions

Genomic technologies challenge aspects of traditional healthcare delivery, with new ethical issues arising from these unchartered waters. The increasing utilization of genomic testing across different healthcare settings over the life cycle necessitates increased clarity of purpose and raises important ethical, legal, and social issues. Healthcare providers will be required to adopt an approach to genomic technology that will allow for the advancement of genomic knowledge and the responsible application of technology to benefit the population across the life cycle. In the context of the complexity and versatility of genomic information and its inherently personal and familial nature, adequate governance and informed consent are critical considerations for implementing genomic testing for healthcare.

## Author Contributions

TW, HD, and GAB conceived the paper concept. BB, GAB, EC, and KN drafted the manuscript. All authors provided critical input and approved submission of the final manuscript.

### Conflict of Interest Statement

The authors declare that the research was conducted in the absence of any commercial or financial relationships that could be construed as a potential conflict of interest.
